# The Use of Adipose-Derived Stem Cells (ADSCs) and Stromal Vascular Fraction (SVF) in Skin Scar Treatment—A Systematic Review of Clinical Studies

**DOI:** 10.3390/jcm10163637

**Published:** 2021-08-17

**Authors:** Albert Stachura, Wiktor Paskal, Weronika Pawlik, Maciej J. Mazurek, Janusz Jaworowski

**Affiliations:** 1 Center for Preclinical Research, Department of Methodology, Medical University of Warsaw, 02-091 Warsaw, Poland; wiktor.paskal@wum.edu.pl; 2 Timeless Plastic Surgery Clinic, 02-091 Warsaw, Poland; maciek.j.mazurek@gmail.com (M.J.M.); jaworowskijanusz@gmail.com (J.J.); 3 Doctoral School, Medical University of Warsaw, 02-091 Warsaw, Poland; 4 Faculty of Medicine and Dentistry, Pomeranian Medical University, 70-204 Szczecin, Poland; weronikapawlik7@gmail.com; 5 West Pomeranian Center for Severe Burns and Plastic Surgery, 72-300 Gryfice, Poland; 6 Plastic Surgery Department, Centre for Postgraduate Medical Education, 02-091 Warsaw, Poland

**Keywords:** stem cells, scar, remodeling, regeneration, extracellular matrix, adipose-derived stem cells, stromal vascular fraction, lipofilling, nanofat

## Abstract

In recent years, lipofilling became a popular scar treatment method. Its beneficial outcomes have been partly attributed to the regenerative capacity of adipose-derived stem cells (ADSCs), suspended in an extracellular matrix—the stromal vascular fraction (SVF). The aim of this review was to verify if existing data support the clinical use of ADSC-related interventions in scar treatment. A systematic search of the literature was performed in July 2020 in five databases (Medline, Cochrane, Web of Science, Scopus and Embase). Articles written in English, except for reviews, letters and editorials, were identified and screened for eligibility. We looked for reports of any outcomes in scars treated with ADSCs or SVF. Data from selected articles were extracted and the quality of each study was assessed. Five hundred and fourteen studies were identified in the primary search, of which nineteen were eventually included in the systematic review. Extracted data pointed to beneficial microscopic, functional and aesthetic outcomes in a total of 665 patients. Six studies included comparative interventions—platelet-rich plasma or CO_2_ fractional laser. Collected data give low-to-average quality evidence for beneficial effects of ADSC-related interventions in scar treatment. Some studies suggest that these interventions are noninferior to PRP or fractional CO_2_ laser.

## 1. Introduction

Skin regeneration after significant injuries comprises subsequent phases, eventually leading to scarring and remodeling. In some cases, however, scars may produce substantial functional disability or distress caused by undesirable aesthetics. To cope with such issues, many treatment modalities have been tried out. For hypertrophic scars, interventions such as silicone, pulsed-dye laser, CO_2_ laser, corticosteroids, 5-fluorouracil, bleomycin and scar massage have high efficacy [1]. Various lasers and PRP (platelet-rich plasma) have emerged as promising scar treatment methods and are assessed in numerous clinical studies. The former approach has been used in different scar types, including atrophic acne scars, showing beneficial effects in small populations [2]. The latter shows promise of enhancing scar quality, especially in surgical scars or in combination with fractional CO_2_ laser or fat grafting [3]. Despite a multiplicity of approaches to cutaneous scar treatment, no gold standard has been established and novel, well-studied methods are still needed.

Autologous fat grafting is an exciting method in plastic surgery and aesthetic medicine, eagerly used in a growing number of indications, one of them being scar treatment [4]. Rigotti et al. demonstrated that much of the desirable outcome of lipofilling is attributed to the regenerative capacity of adipose-derived stem cells (ADSCs), suspended in a fatty tissue cellular matrix—the stromal vascular fraction (SVF) [5]. These cells improve adipogenesis, secrete angiogenic and antiapoptotic factors and may differentiate into multiple cell lineages [5,6,7]. Therefore, it is hypothesized that their higher concentration may produce a more favorable clinical outcome, when used in scar treatment.

Previous systematic reviews concluded that autologous fat grafting seems to have a beneficial effect on scar-related conditions, though the evidence is sparse and of poor quality [8,9,10,11,12]. To date, however, no paper has summarized data on the use of SVF or ADSCs in scar treatment. Physicians who decide to use these cells for clinical or scientific purposes are faced with the challenge of choosing an optimal isolation method—either mechanical or enzymatic. Nanofat—a technique introduced recently by Tonnard et al.—is an attractive alternative to older and usually more complicated SVF isolation protocols [13]. The quantity of SVF cells obtained from nanofat is comparable to enzymatic methods, while requiring less fat tissue intake [14]. We believe a thorough analysis of available methods could be useful for designing future research and possibly shaping clinical practice.

In this review, we aim to gather data from studies reporting the use of ADSCs or SVF in scar treatment, to evaluate the efficacy of such interventions. Moreover, we provide an overview of ADSC/SVF isolation protocols used thus far in clinical studies dedicated to scar treatment.

## 2. Materials and Methods

This study was conducted in accordance with the Preferred Reporting Items for Systematic Reviews and Meta-Analyses (PRISMA) guidelines, using a previously designed protocol (Supplementary File 1) [15]. We searched for studies reporting outcomes of scar or keloid treatments, using ADSCs or SVF isolated from human adipose tissue. All articles written in English, except for reviews, letters and editorials, were included.

We searched using electronic databases: MEDLINE, Cochrane Control Trials Register, EMBASE, Web of Science and Scopus. To identify all relevant articles, we used prespecified search engines for each database (Supplementary File 1). Additionally, we screened references of selected articles to find papers not identified in the primary search. The systematic search of the literature was performed by two independent reviewers in July 2020. Whenever additional information was required, we contacted authors of specific papers via email and/or the ResearchGate website.

### 2.1. Study Selection

Each relevant publication was categorized using the PICO model. Articles were included based on predefined selection criteria: appropriate PICO (using ADSCs or SVF in scar treatment), reporting of the outcomes and a defined isolation protocol. Exclusion criteria were animal studies, review letters and editorials, inadequate PICO, use of artificial materials (e.g., meshes, scaffolds) for delivery of ADSCs or SVF, and a substantial lack of methodology.

Study eligibility was assessed by screening titles and if necessary, abstracts. Later, full texts were assessed for inclusion and exclusion criteria. All disagreements were resolved by a consensus between the two reviewers (A.S. and W.Paskal).

### 2.2. Data Extraction, Risk of Bias and Analyses

The following information was extracted from each study by AS: study design, isolation procedure, population, intervention, microscopic and clinical outcomes. WP assessed the data extraction procedure and implemented necessary corrections. Risk of bias was assessed for randomized controlled trials using Cochrane’s Risk of Bias 2 Tool [16]. Strengths and weaknesses of remaining studies are described separately.

We did not perform quantitative statistical analysis of selected studies because of methodological and clinical heterogeneity. A systematic review of the methodology and outcomes was undertaken instead.

## 3. Results

### 3.1. Search Results

**Figure 1:**
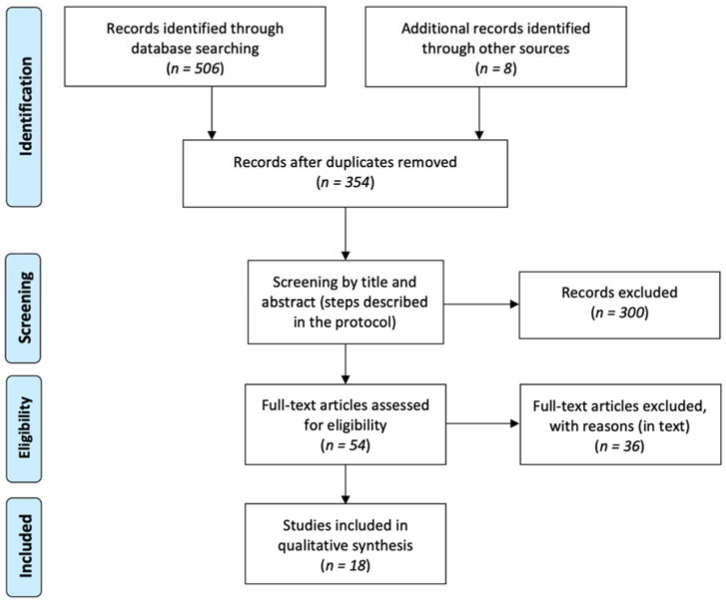
PRISMA flow diagram of search and selection strategy.

The primary search yielded 514 scientific papers (Figure 1). After deduplication, this number was reduced to 354. We performed title and, if necessary, abstract screening. Eventually, full texts of 54 articles were assessed for eligibility. We excluded 36 studies due to: use of normal lipofilling [17,18,19,20,21,22,23,24] (nine), use in other medical conditions [25,26,27,28,29,30,31] (seven), insufficient outcome data [32,33] (three), use of an artificial material for SVF delivery [34,35] (two), inappropriate design [36,37,38] (three), non-English language [39,40,41] (three), ongoing clinical trial [42,43] (two), and use of predifferentiated cells [44] (one). In four cases [45,46,47,48] full texts were unavailable and in two [49,50] only conference abstracts were accessible. We contacted authors of these papers, enquiring about additional data and received only three replies; in all cases authors needed more time to report their findings. Finally, 18 papers (19 studies) were included in this systematic review (Table 1).

### 3.2. Case Reports

Wu et al. [51] reported the correction in scar contour and soft tissue reconstruction in an adherent post-excisional scar on the lower back. The treatment comprised of administering a composition of fat graft, SVF-enriched fat, collagenase and hyaluronic acid beneath the scar. Pain resolved after 6 weeks. At the 3-month follow-up, the lateral scar aspect was corrected 100% and a 77% defect correction on ultrasound examination was reported.

Carstens et al. [53] treated burn scarring in the hand with isolated SVF administered into joints and SVF-enriched lipofilling to the hand dorsum. Six weeks after treatment the patient regained a full range of motion in previously restricted joints—this effect was corroborated at 6, 12 and 24 months, post-op. New blood vessels were detected in the treated areas on ultrasound examination after 4 months. This was the first study showing that SVF injection improved manual functionality in post-burn scarring.

Pallua et al. [68] presented two cases of patients with post-traumatic or post-acne scars treated with microfat, nanofat and PRP. In both cases satisfying aesthetic outcomes were achieved.

The first two cases presented innovative uses of SVF in scar treatment, both incorporating ultrasound examination as an objective measurement tool. Pallua et al. used lipoconcentrate—not an entirely new approach since similar methods have been described earlier. All these studies lacked a statistical analysis (Table 2).

### 3.3. Case Series

Ghareeb et al. [58] treated 30 facial scars (mostly atrophic) with subcutaneous nanofat injections. After 6 months, all VSS components significantly improved (*p* < 0.05), apart from height, which was not reported. Fat resorption occurred in six cases. Seventy-six percent of patients assessed the results as excellent or good. This study showed significant aesthetic improvement in atrophic scars after nanofat injection.

In his second study, Carstens et al. [59] reported five cases of patients with mature burn scars, treated with enzymatically isolated SVF. Scars were localized predominantly on hands, restricting movement. After 6 months, the majority of the treated zones improved (*p* < 0.05) in terms of pigmentation (78,6%), flexibility (100%), thickness (90,4%), pruritus (94%), pain (100%) and vascularity (33%). It is the second study that showed significant improvement in manual functionality after SVF injection in post-burn hand scarring.

Bhooshan et al. [60] used nanofat to treat 34 patients with post-traumatic, post-burn or post-inflammatory scars, the majority of them hypertrophic (82%) and localised on the face (85%). After 3 months, 76.5% had good aesthetic results, which meant a POSAS (Patient and Observer Scar Assessment Scale) score of 6–24, and 23.5% had bad results, which meant a POSAS of >24. All factors evaluated in POSAS improved after treatment (*p* < 0.05), apart from the scar surface area. Of patients with a scar history of <5 years, 92.6% had good aesthetic results, whereas only 14.3% of patients with older scars shared this outcome (*p* = 0.001). In this study, significant aesthetic improvement in mostly hypertrophic scars was shown after nanofat injection.

Gu et al. [61] studied the use of condensed nanofat in 25 atrophic facial scars of various etiology (mostly linear). Clinical outcomes were evaluated with POSAS preoperatively and after 6 months. All variables, measured by patients and physicians, apart from pain, itching and vascularization, improved statistically significantly. Pathological examination showed increased melanin average optical density 0.671 vs. 0.844 (*p* = 0.01), but no changes in elastic fibers. Previously undetectable sebaceous and sweat glands were visualized by immunostaining 6 months after treatment. Here, nanofat rendered significant aesthetic improvement in atrophic facial scars.

Lee et al. [62] reported a case series of 17 patients (19 scars), who received SVF injection alone or in the course of other procedures (scar revision, fat grafting etc.). Treated scars presented a vast spectrum of characteristics (hypertrophied, depressed, contractile, etc.). OSAS, VAS, VSS and SBSES scales were used to assess the clinical outcome. Compared to baseline, OSAS and VSS median scores dropped by five and three, respectively, while SBSES and VAS increased by one and two after 6 months (*p* < 0.01). Vascularity, pigmentation, hardness, flexibility and pliability improved in particular (*p* < 0.01). In this study, scar aesthetics improved after SVF injection, though various assessment methods provided ambiguous results.

Uyulmaz et al. [63] treated scars (undefined characteristics) in 40 patients with nanofat injections. Scar aesthetic improvement was noticeable 100 days after treatment. Three independent doctors reviewed the outcome as good in 74% of the cases and satisfactory in 18% after 3 months. Statistical analysis was not performed. Although beneficial aesthetic outcomes were reported after nanofat injection, no objective scale was utilized to assess the results.

Jan et al. [66] reported a series of 48 patients with post-burn facial scars, treated with nanofat injections. At the six-month follow-up, POSAS improved significantly in all patient-assessed parameters, compared to baseline (*p* < 0.001). In the observer’s opinion, the overall score was better, but only pliability and pigmentation improved significantly (*p* < 0.001). Here, nanofat injections rendered significant aesthetic improvement in post-burn facial scars.

Most studies utilized objective outcome assessment scales, but some failed to address the SVF count or presented significant scar heterogeneity, thus hindering interpretation of the results. A case series by Jan et al. was the strongest. It focused on a big homogenous group of patients (*n* = 48), analysing it prospectively (Table 2).

### 3.4. Case-Control Studies

Gentile et al. [52] studied a group of 30 patients with burn or post-traumatic scars. The control group received a Coleman’s fat graft. In patients, who received SVF- or PRP-enriched fat grafts, scars maintained their contour and volume in 63% and 69%, respectively, compared with the control group (39%) after 1 year (*p* < 0.001). MRI and ultrasound examination showed lower fat reabsorption in the SVF and PRP groups in treated facial scars. In this study, authors showed that SVF- or PRP-enriched fat grafting significantly improved graft survival, thus improving the aesthetic outcome.

The second study by Lee et al. [62] compared two groups of patients who underwent scar revision with (seven patients) or without (eight patients—control) SVF injection. Eleven scars were widened, four hypertrophic and three depressed. After 6 months, the median score in OSAS, VSS and VAS improved in both groups with better outcomes in the study group (*p* < 0.05). Height (*p* = 0.04) for the SBSES and pliability (*p* = 0.04) in the VSS were significantly better in the SVF-treated group vs. control. Here, aesthetics of scars significantly improved after SVF injection compared to no SVF supplementation.

Authors of the first study incorporated objective imaging techniques with an adequate follow-up, however, they failed to report some of the outcomes. This was the first study to compare PRP with SVF-enriched fat in scar treatment. Lee et al. used objective scar assessment scales and masked the analyst, but collected a small and heterogenous sample of patients. Both studies lacked a statistical analysis of patients’ baseline characteristics (Table 2).

### 3.5. Prospective Cohort Studies

Zhou et al. [55] performed a split-face study in 13 patients with facial atrophic acne scars. Three courses of topically applied ADSC-conditioned medium combined with CO_2_ fractional laser were used in monthly intervals on one side of the face. DMEM was applied to the control side. One month after the third treatment, patients’ satisfaction with the ADSC-CM-treated side was significantly higher vs. the control side (2.35 ± 0.69 vs. 2.08 ± 0.76). The result was corroborated by objective assessment with the ECCA (échelle d’évaluation clinique des cicatrices d’acné) score (26.15 ± 19.16 vs. 32.7 ± 18.1), performed by two blinded evaluators. In the ADSC-CM group, the melanin index and transepidermal water loss were significantly lower at the end of the study, whereas biophysical examination showed improved elasticity and hydration. Cheek biopsies showed a more ample improvement in collagen (49.98% vs. 36.09%) and elastin (37.61% vs. 26.13%) density vs. control. In this work, ADSC-conditioned medium rendered aesthetic, biophysical and histological improvement in patients with atrophic acne scars, compared to placebo.

Abou Eitta et al. [64] conducted a split-face study, comparing SVF and CO_2_ fractional laser in the treatment of post-acne scars in 10 patients. Three months after treatment, scar severity decreased in both groups (*p* = 0.004; *p* = 0.005), as measured by the Goodman and Baron scale, with no differences between the cohorts (*p* = 0.183). Scar area percentage was reduced after 2 and 3 months—similarly in both groups (*p* < 0.001). TEWL improved quicker in the SVF-treated group (*p* = 0.004); however, the final TEWL and hydration outcomes were similar on both sides after 3 months (*p* = 0.279). No difference in patients’ satisfaction was noted (*p* = 0.234). The authors showed that there were no significant differences in biophysical and aesthetic outcomes, when comparing SVF with fractional CO_2_ laser in post-acne scars.

Shalaby et al. [67] compared treatment with nanofat vs. nanofat + PRP in 60 patients with atrophic scars of various origin. Total VSS decreased in the control group by a mean of 3.3 and in the study group by a mean of 2.2, with pliability and height improving in both. Results did not differ between cohorts. Patients treated with nanofat + PRP were older and had older scars than those treated with nanofat only. Here, authors showed that PRP supplementation to nanofat is non-superior to nanofat alone, when assessing aesthetic outcomes.

Despite smaller populations, the first two studies utilized more adequate methods than the last one. Their major advantages were an innate control group (split-face), scar homogeneity and a blinded outcome assessment. Baseline differences between the groups and the etiology of various scars hinder interpretation of Shalaby’s study (Table 2).

### 3.6. Randomized Controlled Trials

Elkahky et al. [54] compared enzymatically isolated SVF and PRP treatments in 20 patients with rolling post-acne scars. The mean scar surface reduction percentage increased after 1 month, but did not differ between the groups (*p* = 0.218). However, after 3 months, a higher reduction percentage was shown in the PRP group (80.2%) vs. the SVF group (66.5%) (*p* = 0.023). Authors used an image analyzer to perform a quantitative histological assessment. Epidermal thickness (SVF: 58.5 ± 11.5 vs. 105.8 ± 37.6; PRP: 62.3 ± 5.7 vs. 124.5 ± 21.4) and collagen content (SVF: 15% vs. 25%; PRP: 21% vs. 32%) improved significantly (*p* < 0.05) with no differences between both groups. Elastin was more intensively produced after the PRP treatment (40% vs. 30% in the SVF group, *p* = 0.002). In this study, an advantage of PRP over SVF was shown in terms of long-term scar surface reduction and elastin concentration, but not patients’ satisfaction, nor other histological measurements.

Gentile et al. [56] compared different nanofat procedures in 43 patients with burn or post-traumatic scars. Nanofat modifications included SVF-enrichment and additional mechanical processing steps. Clinical outcomes were assessed after 6 months by patients and operators, by scoring skin quality factors on a scale from zero to five. Best results were obtained in the supercharged nanofat group (means 25.6; 25.7), followed by evo (25.3; 25.2), centrifuged (24; 23.8) and classic (22.6; 22) nanofat with significant differences between subsequent cohorts (*p* < 0.05). SVF yields were measured and compared between the groups. Authors associated the SVF cell number with clinical improvement. Here, nanofat enhanced aesthetics in patients with post-burn or post-traumatic scars, with improvement proportional to the degree of nanofat condensation.

Tenna et al. [57] studied CO_2_ fractional laser addition to nanofat + PRP treatment in a group of 30 patients with chronic acne scars. All patients underwent two courses of either treatment (with or without laser) 6 months apart. Three months after the second treatment, skin thickness improved in the laser-treated group (*p* = 0.007), but not in control (*p* = 0.12), compared with the preoperative values. The calculated change in thickness between the pre- and post-op period, however, did not differ between groups (0.67 cm in A and 0.63 cm in B). Baseline skin thickness differed between groups (0.532 cm in A and 0.737 cm in B). Measurements were taken with ultrasound. The postoperative patients’ quality of life was similar in both groups, evaluated with the FACE-Q module. Results of this study are not easy to interpret due to significant baseline imbalances between the groups. It seems fractional CO_2_ laser addition to nanofat + PRP may improve skin thickness, but the credibility of this finding is questionable.

Malik et al. [65] treated 10 patients with painful amputation stumps with SVF-enriched fat grafting or fat grafting alone. Both the overall score and individual parameters of POSAS significantly decreased 1 and 6 months after the treatment (*p* < 0 05), similarly in both groups. Compared to baseline, postoperative MRI scans showed increased fat accumulation over the stump in the SVF-treated groups, but not in the control. The authors showed that SVF supplementation prolongs the fat graft survival, though it does not improve aesthetic outcomes compared to fat grafting alone.

The above-mentioned RCTs were of poor quality, bearing a considerable or high risk of bias (Table 3). Only Malik et al. properly described the randomization process. All studies lacked information about patient, physician and outcome assessor blinding. Baseline characteristics were adequately analyzed in the first and fourth study. Authors of the second study provided basic information about studied groups but performed no statistical analysis. They also selectively reported outcomes, missing data from the 12-month follow-up, declared in the methods.

### 3.7. Isolation Protocols

**Figure 2:**
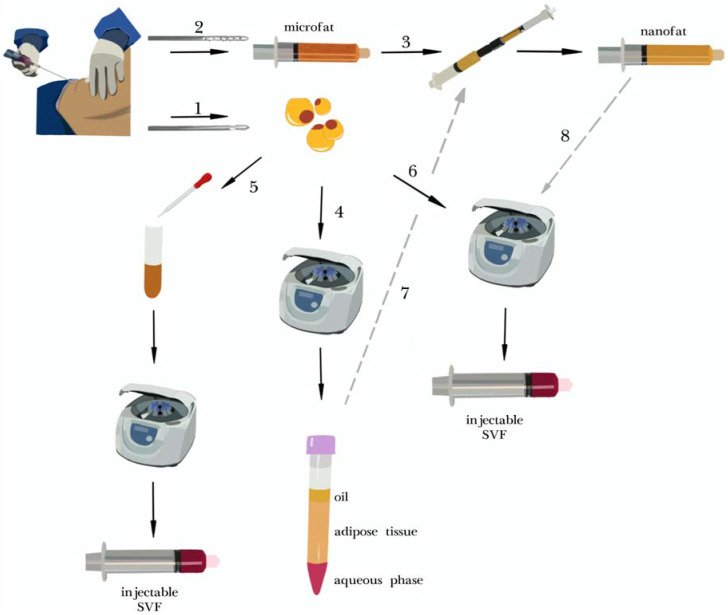
Overview of fat processing and stromal vascular fraction (SVF) isolation techniques. Liposuction can be performed either with a classic cannula (**1**) for normal fat harvesting or with a multi-perforated cannula (**2**), which results in obtaining microfat. If microfat is subsequently processed 30 times between two Luer-Lock syringes (**3**), the emulsified product is called nanofat. Centrifugation (**4**) is used to condensate fatty tissue (most commonly with Coleman’s protocol at 3000 RPM for 3 min) [69]. The stromal vascular fraction (SVF) may be obtained by enzymatic isolation (**5**) with the use of collagenase and subsequent centrifugation. Alternatively, mechanical isolation may be used (**6**) usually it comprises several steps of mechanical dissociation of fatty tissue with centrifugation, filtration etc. Some researchers combine prior centrifugation with mechanical emulsification, thus producing more condensed nanofat (**7**). Another modification may be additional centrifugation after nanofat production (**8**).

Authors of selected articles used a variety of isolation techniques. The majority of them are illustrated in a simplified form in Figure 2; all details are described in Supplementary Table S1. The most commonly used procedures were enzymatic SVF isolation [52,53,54,55,59,62,64,65] and nanofat [56,57,58,60,61,63,66,67,68]. Wu et al. used spectroscopy for SVF isolation [51]. The fatty tissue was harvested from the abdomen (seven) or multiple sites (seven), including flanks, hips or thighs. The liposuction site was not specified in four cases.

Protocols of enzymatic SVF isolation varied between studies. Most commonly (six studies) they included tissue digestion with collagenase and subsequent centrifugation (steps one and five in Figure 2). Lee et al. condensed the fat prior to enzyme addition. Zhou et al. cultured the ADSC fraction after isolation and eventually produced a cell-free medium, rich in growth factors and cytokines, later used in the study.

Nanofat was used in nine cases. Its production, however, differed between studies. In six cases, fat condensation was performed prior to mechanical emulsification (steps four and seven, Figure 2). In two studies nanofat was additionally centrifuged afterwards (step eight). In three cases nanofat was produced in a classic way (steps two and three, Figure 2). Gentile et al. produced three modified versions of nanofat—enriching it with mechanically isolated SVF (step six) or performing additional mechanical processing.

## 4. Discussion

In this review, we gathered information from 19 studies, reporting outcomes in a total of 665 patients. The majority of studies (10/19) are case reports or case series. Among randomized controlled trials, the overall risks of bias were assessed as considerable or high. Nevertheless, authors present corroborating results, suggesting beneficial effects of ADSCs/SVF in scar treatment.

Internationally accepted scar quality measuring tools like VSS, POSAS or VAS enable simple and objective outcome evaluation [70] and were used in nine studies [55,58,59,60,61,62,65,66,67]. They unanimously reported clinical improvement after nanofat/SVF administration. Other authors also emphasized descriptive satisfying results in terms of scar texture, colour, softness, elasticity, vascularization and hydration after these interventions. SVF was used with success in the treatment of six cases of hand burns by Carstens et al., facilitating the rehabilitation process [53,59].

Few studies compared nanofat/SVF with alternative treatments. Results suggest that aesthetic outcomes and patients’ satisfaction do not differ significantly in comparison with classic fat grafting. Fat accumulation and reabsorption, however, improves in nanofat/SVF-treated groups [52,65]. Three studies compared PRP with SVF/nanofat and suggested improved scar area reduction in the PRP group with little or no differences in fat resorption, clinical or microscopic findings [52,54,67]. However, due to the considerable risk of bias in these reports, the superiority of either method cannot be clearly stated. Abou Eitta et al. prospectively compared SVF with fractional CO_2_ laser in the treatment of post-acne scars but found no significant differences [64].

Quantitative and semiquantitative histological analyses undertaken in selected studies showed increased elastin and collagen production, coupled with increased dermal thickness and neovascularization [54,55,56,61]. Gu et al. visualized sebaceous and sweat glands, usually absent or scarce in scars, 6 months after nanofat injection. Zhou et al. showed that topically administered ADSC-conditioned medium improves the alignment of fibers.

The abundance of SVF/ADSC isolation methods and multiple nanofat processing protocols pose a challenge to interpreting collected results. They also reflect great heterogeneity of clinical practices. Some authors altered the original nanofat production procedure by additional centrifugations before and/or after the homogenization step [56,57,58,61,67,68]. In vitro studies demonstrated that similar modifications increase ADSCs output [33,71,72,73], without affecting the composition of secreted proteins [73]. However, it remains unclear whether higher stem cell yields translate into clinical improvement. This subject needs to be studied carefully. Gentile et al. showed only a post-hoc association between ADSC’s quantity and clinical outcomes. However, the significance of this observation, may be undermined by a relatively small study population and a high risk of bias [56].

This review is limited by English language preference and the exclusion of animal studies (possibly including human subjects) from the search engine. We are awaiting reports of randomized trials studying preventive SVF use, keloid treatment, SVF comparison with PRP and isolated ADSC administration [42,43,45,46,47,48].

## 5. Conclusions

Collected data give limited low-to-average quality evidence for beneficial effects of ADSC-related interventions in scar treatment. Some evidence suggests that SVF/nanofat is noninferior to common approaches, such as PRP or fractional CO_2_ laser in terms of clinical outcomes. Many poor-quality papers were published and high-quality data are needed to support the use of ADSCs/SVF in clinical practice. Adequate randomized controlled trials are required to compare ADSC-related interventions with other methods, as well as different ADSC/SVF isolation methods with each other. Hopefully, this review will pave the way for conducting future research and will be helpful in navigating through methodological discrepancies.

## Figures and Tables

**Table 1:** Summary of design, SVF/ADSC isolation procedures, populations, interventions, clinical and microscopic outcomes of selected studies.

Reference	Study Design (Level of Evidence)	SVF/ADSC Isolation Technique	No. of Patients (Scars)	Scar Types	Treatment (Study Groups)	Clinical/Macroscopic Outcomes	Microscopic Findings
Wu et al. 2013 [51]	Case report (V)	SVF: Gravitational decanting -> Coleman’s procedure -> spectroscopy	1	cicatrix scar after lipoma excision on the back	PALF with SVF-enriched autologous fat transfer in conjunction with collagenase and hyaluronic acid serum with trichloroacetic acid peeling	Resolution of scar adherence against the muscle, reduced swelling, quicker epithelialization, improvement in clinical (texture, color, size) and ultrasonographic examination, and pain resolution *	None were examined due to patient’s lack of consent.
Gentile et al. 2014 [52]	Case-control (III)	SVF: Enzymatic isolation with a commercially available system	30	burn or post-traumatic scars	1. Coleman’s fat graft (control) 2. SVF-enriched autologous fat graft 3. Coleman’s fat graft + PRP	Contour restoring and volume maintenance improvement (39%—control, 63%—SVF, 69%—PRP)†, lower fat reabsorption in facial scars in study groups vs. control *. Patients’ satisfaction with texture, softness and contour in all groups *.	None were examined.
Carstens et al. 2015 [53]	Case report (V)	SVF: Enzymatic digestion -> centrifugation	1	fibrosis of the right hand as late sequelae of a burn scar	Local injections of isolated SVF into 4 MCP joints and SVF-enriched lipofilling of the dorsum of the hand	Range of motion restoration in MCP, PIP and DIP joints, full opposition of the thumb after 6 weeks. Improvement of skin color and elasticity. Increased vascularization *	None were examined.
Elkahky et al. 2016 [54]	Low-quality randomized controlled trial (II)	SVF: Enzymatic digestion -> centrifugation -> filtering	20	atrophic rolling facial post-acne scars	1. Intradermal injection of SVF 2. Intradermal injection of PRP underneath the scars on the entire face	Total scar surface area reduction after 1 month (no difference between groups) and 3 months (66.49 ± 12.82—SVF vs. 80.2 ± 8.9 in the PRP group †). Patients reported high satisfaction rates and good treatment tolerance *.	At 3 month follow-up, increased epidermal thickness, number and density of collagen and elastic fibers †, redevelopment of rete processes, acanthosis, spongiosis
Zhou et al. 2016 [55]	Prospective cohort study (II)	ADSC-CM: Enzymatic digestion -> centrifugation -> filtering -> centrifugation -> cell culture -> conditioning in hypoxia -> medium collection	13	Facial atrophic acne scars	Split-face study: 1. 3 × FxCR + topical DMEM (control) 2. 3 × FxCR + topical ADSC-CM	Higher patients’ satisfaction (2.35 ± 0.69 vs. 2.08 ± 0.76) and clinical improvement in the study group, measured with ECCA score (32.69 ± 18.1 vs. 26.15 ± 19.16) †. Lower melanin index and TEWL; higher elasticity and hydration in ADSC-CM-treated group †.	Semi-quantitative analysis showed increased collagen (49.9 ± 0.6% vs. 36.1 ± 0.6%) and elastin (37.6 ± 0.8 vs. 26.1 ± 0.4%) density†, more orderly alignment of fibers in ADSC-CM treated sample *.
Gentile et al. 2017 [56]	Low-quality randomized controlled trial (II)	Nanofat: 1. Mechanical dissociation -> filtering 2. Mechanical isolation of SVF with commercially available system + nanofat 3. Centrifugation -> mechanical fat dissociation 4. Low-speed centrifugation -> mechanical fat dissociation	43	burn or post-traumatic scars	Intradermal injections: 1. Nanofat (control) 2. Supercharged nanofat (SVF-enriched nanofat) 3. Centrifuged nanofat 4. Evo nanofat	Scoring of pigmentation, vascularization, pliability, thickness, itching and pain by the patients and operator showed that supercharged (means 25.6; 25.7) > evo (means 25.3; 25.2) > centrifuged (means 24; 23.8) > classic nanofat (means 22.6; 22) †.	Significant improvement of epidermal and dermal thickness in all studied groups after 6 months with no difference between them†. New collagen and vessels formation in a representative sample from the supercharged group *.
Tenna et al. 2017 [57]	Low-quality randomized controlled trial (II)	Nanofat: Coleman’s procedure -> mechanical emulsification	30	Chronic acne scars	Two treatments (6 months interval) with either (subcutaneous injections): 1. Nanofat + PRP (control) or 2. Nanofat + PRP +FxCR	Significant improvement of skin thickness 3 months after the 2nd treatment in group 2 (0.74 cm vs. 1.37 cm) †, but not in control (0.53 cm vs. 1.2 cm), compared with baseline. No difference in skin thickness increase between the groups.	None were examined.
Ghareeb et al. 2017 [58]	Case series (IV)	Nanofat: Coleman’s procedure -> mechanical emulsification	30	Facial scars—various etiology—26 were atrophic	Subcutaneous nanofat injections	Significant improvement in scar vascularity, pigmentation, pliability and pruritus as per VSS score †. Satisfaction in 76% of the patients.	None were examined.
Carstens et al. 2017 [59]	Case series (IV)	SVF: Washing -> enzymatic digestion -> centrifugation	5 (35 treatment zones)	Burn scars	Subcutaneous nanofat injections	Significant improvements in VSS score, scar hardness (durometer), elasticity (cutometer) † and patients’ satisfaction *.	None were examined
Bhooshan et al. 2018 [60]	Case series (IV)	Classic nanofat	34	Post-traumatic, burn or post-inflammatory scars	Nanofat injected intralesionally	Significant improvement in POSAS—mean 27.4 ± 7.5 vs. 14 ± 14.4 (patient’s assessment) and mean 31 ± 8.5 vs. 18 ± 6.8 (observer’s assessment). Significantly better results in younger scars (<5 years) †	None were examined.
Gu et al. 2018 [61]	Case series (IV)	Nanofat: Coleman’s procedure -> mechanical emulsification -> centrifugation (3000 RPM × 3min)	20 (25)	Atrophic facial scars (post-surgical, burn, post-traumatic and post-acne)	Condensed nanofat intradermal injection. One scar required additional subcutaneous lipofilling.	Significant clinical improvement both in patient’s (28.8 ± 1.02 vs. 12.2 ± 0.8) and physician’s (18 ± 0.71 vs. 9.2 ± 0.37) assessment, measured with a POSAS score†.	6 months post-op: increased melanin density (0.671 vs. 0.844) †. Sebaceous and sweat glands visualized with CK14 and CK19 staining.
Lee et al. 2018 (two studies) [62]	Case series (IV)/Case-control (III)	SVF: Centrifugation -> enzymatic digestion -> multiple centrifugations	Study 1: 17 (19) Study 2: 15	Various; restricted to face in study 2.	Study 1: SVF injection (s.c./i.d.) alone or in the course of other procedures. Study 2: Scar revision with or without SVF injection (s.c./i.d.) (2 groups)	Study 1: Improvement of OSAS (vascularity, pigmentation, hardness, flexibility), SBSES (only in overall score), VSS (vascularity, pigmentation, pliability) and VAS median scores 6 months post-op vs. baseline† Study 2: Improvement of OSAS, VSS and VAS overall median scores, as well as height and pliability in the SVF-treated group vs. control after 6 months †.	None were examined.
Uyulmaz et al. 2018 [63]	Case series (IV)	Classic nanofat	40	Various	Nanofat injection into scars or i.d. (twice in 4 cases)	Softer and less prominent scars. Good or satisfactory clinical outcome in most cases. Improved patients’ satisfaction *	None were examined.
Abou Eitta et al. 2019 [64]	Prospective cohort study (II)	SVF: Washing -> enzymatic digestion -> centrifugation	10	post-acne scars	Split-face study: 1st half—intradermal SVF injection 2nd half—3 × FxCR	At 3 month follow-up, significant reduction in scar severity and area percentage compared to baseline. TEWL, hydration, patients’ satisfaction, skin texture and homogeneity improved. No differences between the groups.	None were examined.
Malik et al. 2019 [65]	Low-quality randomized controlled trial (II)	SVF: Gravitational decanting -> enzymatic digestion -> centrifugation	10	amputation stump scars	Injection into scarred stump: 1. Fat grafting (control) 2. SVF-enriched fat graft	After 6 months, POSAS overall score (mean sum of 77 vs. 40.4 in cases and 79.2 vs. 42,4 in controls) and all its individual parameters improved over time in both groups †. Fat accumulation over stump increased in SVF-treated (mean fat area 17.9 vs. 26.8) patients †, but not in control (24.1 vs. 28.8).	None were examined.
Jan et al. 2019 [66]	Case series (IV)	Classic nanofat	48	Post-burn facial scars	Nanofat injection (subcutaneous or intradermal)	After 6 months, improvement of POSAS score in all patient-measured parameters + pigmentation and pliability, measured by observer (overall observer’s mean 7.5 ± 0.77 vs. 4.33 ± 0.48) †	None were examined.
Shalaby et al. 2020 [67]	Prospective cohort study (II)	Nanofat: Coleman’s procedure -> mechanical emulsification (90×)	60	Atrophic scars	Intradermal and subcutaneous injections of either: 1. Nanofat (control) 2. Nanofat + PRP	After 3 months—significant improvement in scar pliability, height and total VSS score (4.6 ± 1.7 vs. 2.4 ± 1.3 in nanofat + PRP; 5.2 ± 1.8 vs. 1.9 ± 1.4 in nanofat group), but no differences between the groups †.	None were examined
Pallua et al. 2020 [68]	Case reports (V)	Nanofat: Centrifugation -> mechanical emulsification -> cenrifugtion	2	Post-traumatic or post-acne facial scars	Subcutaneous microfat injection + s.c./intradermal nanofat injection ± PRP	Improvement in skin and scar quality, improved flexibility and decreased irritation. 6 months–1 year follow-up *	None were examined

SVF—stromal vascular fraction, ADSC—adipose-derived stem cells, PALF—percutaneous aponeurotic lipofilling, Coleman’s fat graft—as described in Figure 2, PRP—platelet-rich plasma, MCP—metacarpophalangeal, PIP—proximal interphalangeal, DIP—distal interphalangeal, FxCR—fractional carbon dioxide resurfacing, DMEM—Dulbecco’s modified eagle medium, ADSC-CM—adipose-derived stem cells-conditioned medium, ECCA—échelle d’évaluation clinique des cicatrices d’acné, TEWL—transepidermal water loss, POSAS—patient and observer scar assessment score, OSAS—observer scar assessment score, VAS—visual analog scale, VSS—Vancouver scar scale, mVSS—modified Vancouver scar scale, s.c.—subcutaneous, i.d.—intradermal. * no statistical analysis. † statistically significant outcome (*p* < 0.05).

**Table 2:** Strengths and weaknesses of non-randomized trials.

Reference	Study Design	Strengths	Weaknesses
Wu et al., 2013 [51]	Case report	One of the first studies describing SVF use in scars Ultrasound imaging performed Innovative use of SVF	Lack of control group or statistical analysis Lack of SVF count and patient’s age
Carstens et al., 2015 [53]		Functional outcomes assessment Ultrasound imaging performed One of the first SVF uses in scars Adequate follow-up (24 months)	Lack of control group or statistical analysis Incomplete fat harvesting data Injection technique not specified
Pallua et al., 2020 [68]		Use of condensed nanofat with increased number of ADSCs Adequate follow-up (6–12 months)	Lack of control group or statistical analysis Lack of SVF count and patients’ age
Ghareeb et al., 2017 [58]	Case series	Scar assessment scale used (VSS) Sufficient statistical analysis	Lack of control group Lack of SVF count and scars’ age Scars resulting from various injuries (post-traumatic, post-burn, post-inflammatory) Heterogeneity of scars’ characteristics *
Carstens et al., 2017 [59]		Scar assessment scale used (VSS) Prospective design Homogenous (post-burn) scar group Multiple outcomes assessed (hardness, elasticity, range of motion)	Lack of control group Small study population (*n* = 5) Incomplete fat harvesting data
Bhooshan et al., 2018 [60]		Scar assessment scale used (POSAS) Prospective design Sufficient statistical analysis Scar age included in the analysis (5 years cut-off)	Lack of control group Heterogeneity of scars’ characteristics* Subjective threshold for aesthetic result assessment Incomplete fat harvesting data Lack of SVF count
Gu et al., 2018 [61]		Scar assessment scale used (POSAS) Homogenous (facial atrophic) scar group Prospective design Histological analysis, including various staining methods and immunohistochemistry Sufficient statistical analysis and a detailed report of used methodology	Lack of control group Scars resulting from various injuries (surgical, post-burn, traumatic) Lack of SVF count
Lee et al., 2018 (1st study) [62]		Scar assessment scales used (OSAS, SBSES, VSS, VAS) Blinded outcome assessment Statistical analysis was performed	Lack of control group Heterogeneity of scars’ characteristics * Interference with additional procedures (scar revision, fat grafting etc.) No confidence interval for presented results Incomplete fat harvesting data
Uyulmaz et al., 2018 [63]		Outcome assessment by three independent specialists	Lack of control group No objective clinical scores No statistical analysis
Jan et al., 2019 [66]		Homogenous (post-burn facial) scar group Prospective design A significant study population (*n* = 48) Scar assessment scale used (POSAS) Sufficient statistical analysis and a detailed report of used methodology	Lack of control group
Gentile et al., 2014 [52]	Case-control	MRI and ultrasound imaging Statistical analysis was performed Adequate follow-up (mean 60 months) Nucleated cells yield reported Novel comparison of SVF-enriched graft with PRP and normal fat grafting	Selective outcome reporting (no results of team evaluation and patient self-evaluation) No statistical analysis of baseline groups’ characteristics Incomplete fat harvesting data No information about scars’ age
Lee et al., 2018 (2nd study) [62]		Scar assessment scales used (OSAS, SBSES, VSS, VAS) Blinded outcome assessment Statistical analysis was performed	Heterogeneity of scars’ characteristics * Small study population (*n* = 15) No statistical analysis of baseline groups’ characteristics Incomplete fat harvesting data No confidence interval for presented results
Zhou et al., 2016 [55]	Prospective cohort studies	Split-face study with inner control group Homogenous (facial atrophic post-acne) scar group Blinded outcome assessment by two investigators Scar assessment scale used (ECCA) Biophysical and histological analyses performed	Small study population (*n* = 13) P-values not reported for individual outcomes
Abou Eitta et al., 2019 [64]		Split-face study with inner control group Homogenous scar etiology (post-acne) SVF identification and cell count reported Blinded outcome assessment Sufficient statistical analysis Multiple clinical outcomes assessed (acne grading, skin function, scar area etc.) Comparison between SVF and FxCR	Small study population (*n* = 10) No information about scars’ age Ambiguous and incomplete description of injection technique
Shalaby et al., 2020 [67]		Scar assessment scale used (VSS) Homogenous (atrophic facial) scar groups A significant study population (*n* = 60) Comparison between nanofat and nanofat + PRP Sufficient statistical analysis	Scars of various etiology Untested nanofat processing technique (90 passes between Luer-Lock syringes) Significant baseline imbalances between groups Lack of SVF count

* more than two scar types, e.g., hypertrophic, atrophic, depressed, widened etc.

**Table 3:** Risk of bias assessment in randomized controlled trials.

	Randomization Process	Deviation from the Intended Interventions	Missing Outcome Data	Measurement of the Outcome	Selective Reporting	Overall Risk of Bias
Elkahky et al., 2016 [54]	?	?	√	?	?	?
Gentile et al., 2017 [56]	?	?	√	×	×	×
Tenna et al., 2017 [57]	×	?	√	×	×	×
Malik et al., 2019 [65]	?	?	√	?	?	?

√—low risk of bias; ×—high risk of bias; ?—some concerns, assessed with Cochrane’s Risk of Bias Tool 2.

## Data Availability

The data supporting the findings of this study are available from the corresponding author, A.S., upon reasonable request.

## Supplementary Materials

The following are available online at https://www.mdpi.com/article/10.3390/jcm10163637/s1, Table S1: Detailed methodology of selected studies; systematic review protocol. Supplementary File 1: Systematic review protocol.
