# Supplementary material for: The Use of Adipose-Derived Stem Cells (ADSCs) and Stromal Vascular Fraction (SVF) in Skin Scar Treatment—A Systematic Review of Clinical Studies
Source: J Clin Med. 2021 Aug 17;10(16):3637. doi: 10.3390/jcm10163637 (PMC8396936; doi:10.3390/jcm10163637)
Supplement: Supplementary file 1 [file jcm-10-03637-s001.zip › File 1.pdf]

Systematic review protocol

PICO

- Population – patients with scars or keloids (animal studies excluded)
- Intervention – **ADSCs** (adipose-derived stem cells) or **SVF** (stromal vascular fraction) use in **skin scar treatment** (including prevention, if well-documented as an outcome/not wound treatment)
- Comparison – none OR control group with placebo OR control group with any other validated and commonly used treatment
- Outcome – any (i.e. symptoms, pain control, scar size or quality etc.)
- Study design – all except for reviews, letters and editorials, English language only

Draft title: Use of adipose-derived stem cells (ADSCs) and stromal vascular fraction (SVF) in skin scar treatment – a systematic review

SUMMARY

Context: Data regarding the use of ADSCs and SVF in scar treatment has not been systematically reviewed and analyzed.
Objective: To gather all available evidence on the effectiveness of ADSCs/SVF in scar treatment.
Data sources: English articles from MEDLINE, Cochrane Control Trials Register, EMBASE, Web of Science and Scopus
Study selection: vide Study design in PICO
Data extraction: Data extraction from articles by AS, followed by a review by W. Paskal, using predefined data fields.
Data synthesis: non-applicable
Conclusions: Collected data give substantial, though average-quality evidence for beneficial effects of ADSCs-related interventions in scar treatment – both clinically and on a microscopic level.

Search engines

Population: "scar" or "scars" or "keloid" or "keloids" NOT animals [mh]
Intervention: "SVF" or "stromal vascular fraction" or "ADSC" or "adipose-derived mesenchymal stem cells" or "adipose-derived stem cells" or "ASC" or "adipose stem cells" or "adipose stromal cells" or "nanofat"

Final (used for Medline, Cochrane and Web of Science)**:** ("scar" or "scars" or "keloid" or "keloids") AND ("SVF" or "stromal vascular fraction" or "ADSC" or "adipose-derived mesenchymal stem cells" or "adipose-derived stem cells" or "ASC" or "adipose stem cells" or "adipose stromal cells" or "nanofat") NOT animals [mh]

- **Medline (PubMed)**
- **Cochrane Library** (term "NOT animals [mh]" not included)
- **Embase** (uses Emtree instead of MeSH terms)

('scar'/exp OR 'scar' OR 'keloid'/exp OR 'keloid') AND ('vsf' OR 'vascular stromal fraction' OR 'nanofat' OR 'adipose derived stem cell'/exp OR 'adipose derived stem cell' OR 'adsc') NOT ('animal experiment'/exp OR 'animal experiment') AND ([article]/lim OR [article in press]/lim OR [conference abstract]/lim OR [conference paper]/lim) AND [english]/lim

- **Scopus (Elsevier)**

TITLE-ABS-KEY (("scar" OR "scars" OR "keloid" OR "keloids") AND ("SVF" OR "stromal vascular fraction" OR "ADSC" OR "adipose-derived mesenchymal stem cells" OR "adipose-derived stem cells" OR "ASC" OR "adipose stem cells" OR "adipose stromal cells" OR "nanofat")) AND (EXCLUDE (EXACTKEYWORD , "Nonhuman"))

- **Web of Science** (term "NOT animals [mh]" not included, adjusted with articles only)

Search strategy and verification

Step 1. Duplicates removal

Step 2. Screening by title:
- Is this a human study?
- Is the study design appropriate?
- Is the subject of this study the use of ADSCs or SVF in scar treatment?

If 3 x "yes" → Study included in step 3
If at least 1 "no" → Study excluded
If not certain → Abstract screening

Step 3.
- Verification of inclusion and exclusion criteria via full text screening

Inclusion criteria:
- Appropriate PICO
- Defined isolation protocol
- Reported outcomes

Exclusion criteria:
- Inadequate PICO
- Lacks in methodology – isolation protocols, follow-up information
- Use of stem cells other than these deriving from adipose tissue
- Use of artificial materials (e.g. meshes) for delivery of ADSCs or SVF
