# Supplementary material for: The Use of Adipose-Derived Stem Cells (ADSCs) and Stromal Vascular Fraction (SVF) in Skin Scar Treatment—A Systematic Review of Clinical Studies
Source: J Clin Med. 2021 Aug 17;10(16):3637. doi: 10.3390/jcm10163637 (PMC8396936; doi:10.3390/jcm10163637)
Supplement: Supplementary file 1 [file jcm-10-03637-s001.zip › Table S1.pdf]

Supplementary Table S1. Detailed methodology of selected studies

Reference	Harvest	SVF/ADSC isolation	SVF yield	Administered solution	Administration route to recipient site	Cannulas/needles	Age of patients	Age of scar(s)	Follow-up period
Wu et al. 2013 [50]	**Site**: anterior abdominal<br>**Infiltration**: standard Klein solution<br>**Technique**: 2 mm inner diameter cannula (Mentor BENSAT 330). **Negative pressure** limited to 350 mmHg.<br>**Volume**: N/A	1. Lipoaspirate washed three times with saline<br>2. Gravitational decanting – aqueous phase removed<br>3. 1000 RCF, 3 min<br>4. Decantation of oil and aqueous phase<br>5. Spectroscopy (EP2346989 A1) to isolate SVF-rich fat	N/A	100+60 ml SVF. Subsequently 0,5ml autologous serum + 1ml hyaluronic acid (HA) filler; 0,1 PZ-U/mL NB6 collagenase	Subdermal, 100 ml, reverse injections, 3-5 ml per pass layering thin ribbons of graft to reach 20% overcorrection: Serum + HA – cross hatched manner - ring around and underneath the scar subdermally; collagenase intradermally	SVF: blunt spatulated tip<br>HA + serum: 22G spinal needle<br>Collagenase: 25G needle	N/A	2 yo	days 1–5 and 1, 2, 4, 8 and 12 weeks
Gentile et al. 2014 [60]	N/A	A) SVF-enriched fat.<br>1. Commercial Celution System used.<br>2. Additional wash and centrifugation cycles,<br>3. 5 mL of the enriched fat extracted<br>4. Added to harvested fat graft<br>B) Coleman's fat mixed with 0.5 mL of PRP. Fat subjected to centrifugation at 3000 RPM; AFT:PRP = 1ml:0,5ml<br>C) Coleman's fat	50 000-250 000 nucleated cells per milliliter of fat (automated count – manual count)	Group A: SVF-enriched fat grafts (up to 10mL, of which 5mL of SVF)<br>Group B: 1mL of Coleman's fat + 0,5 mL PRP<br>Control group: Coleman's fat	A) Subdermally<br>B) Subdermally, small tunnels were previously created, cannulas with 1.5 mm diameter	N/A	21 - 69 yo	N/A	1 y (quantitative data report); mean 60 months
Carstens et al. 2015 [51]	**Site**: N/A<br>**Infiltration:** N/A<br>**Technique**: directly into a sterile processing canister (GID SVF-1)<br>**Negative pressure:** N/A<br>**Volume**: 108 ml dry fat	1. Washed three times with saline<br>2. 125 mL of lactated Ringer's + collagenase enzyme 200CDU/mL<br>3. Incubation – 40 min, 38 °C, 150 RPM	>4x10^{7} mononuclear cells (5x10^{5}/g of dry fat)	1) 2mL of SVF containing a total of 6 x 106 SVF-derived cells<br>2) 20mL of SVF enriched	1) divided and injected into 4 MCP joints<br>2) into subcutaneous space of the	N/A	58 yo	4 yo	6 weeks; 6, 12 and 24 months

		4. Centrifugation for 10 min at 800g<br>5. Removal of SVF from the bottom of the device		with a total of 2.7 x 106 SVF-derived cells	dorsum of the hand				
Elkahky et al. 2016 [64]	**Site**: abdomen<br>**Infiltration**: modified Klein's solution.<br>**Technique**: blunt-tipped cannula<br>**Negative pressure**: N/A<br>**Volume**: 250ml	1. Washed 4-6 times with PBS<br>2. Digestion with 0.2% collagenase at 37 °C for 60 min with agitation<br>3. DMEM addition to inactivate collagenase<br>4. Centrifugation at 1500 RPM for 5 min.<br>5. Cellular pellet resuspension in fetal bovine serum and passing through 100um filter to remove debris<br>6. SVF collection<br>7. SVF was photoactivated using the AdiLight1 System (Adistem Pty) for 15 min.	7 to 86 x 10^6 cells/sample	SVF resuspended in 10% fetal bovine serum	Intradermal injections	N/A	AT-ASC :20 to 43 years (mean: 26.3 ± 7 years)<br>PRP group: 20 to 44 years (mean: 28.7 ± 5 years)	N/A	1 and 3 months
Zhou et al. 2016 [61]	Details N/A, lipoaspiration from 2 subjects (allogenic)	1. Lipoaspirate digested with 0,75% collagenase type II under gentle agitation for 45 min at 37°C<br>2. Centrifugation at 300g for 10 min<br>3. Pellet filtered with a 70um nylon mesh filter<br>4. Resuspension in PBS<br>5. Centrifugation at 840g for 10 min<br>6. Supernatant discarded<br>7. Cell fraction cultured overnight at 37 °C/5% CO_2 in culture medium<br>8. Cell population maintenance for 3-5 days until confluence<br>9. Medium changed to PBS free DMEM<br>10. Exposition to hypoxia (2% O_2/5% CO_2 and balanced N_2) for 72 hours	N/A	3 mL of ADSC-CM	topically applied onto fractional laser-treated sites	N/A	24 to 50 years (mean age 36.4)	N/A	1 month after the last treatment session

		11. Centrifugation at 300g for 5 min<br>12. Filtered using 0.22 mm syringe filter							
Gentile et al. 2017 [65]	**Site**: N/A<br>**Infiltration**: N/A<br>**Technique**: Coleman cannula 3 mm-diameter with side sharp holes of 1 mm in diameter, luer-lock syringes<br>**Negative pressure**: no high negative pressure (no details)<br>**Volume**: 80ml	A) classic mechanical emulsification – 30 passes.<br>B) 160 ml – divided into 1st and 2nd part<br>a. 1st part –<br>i. Automatic filtration (120um filter)<br>ii. Centrifugation at 1300 RPM for 10 min.<br>iii. SVF pellet collection and SVF suspension filtered (120um filter) to final 20 ml<br>b. 2nd part was classically emulsified (30 passes)<br>c. Mixing 1st:2nd 0,2:1<br>C) lipoaspirate was centrifuged at 1300 RPM for 10 min, SVF pellet was discarded, remaining fat layer was emulsified (30 passes)<br>D) lipoaspirate slow centrifugation at 80g for 3 min with Adipecons in 16 ml stocks, further mechanical emulsification (30 passes)	Mean of 5 samples:<br>A. Classic nanofat 20,000 ± 3000<br>B. Supercharged nanofat 200,000 ± 15,000<br>C. Centrifuged modified nanofat 53,334 ± 8000<br>D. Evo-modified nanofat 125,000 ± 12,000	A. Classic nanofat<br>B. Supercharged nanofat<br>C. Centrifuged modified nanofat<br>D. Evo-modified nanofat	Intradermal injections	27G	20-73 yo	N/A	3, 4, 6 and 12 months
Tenna et al. 2017 [66]	**Site**: lower abdomen, flanks, hips and thighs **Infiltration**: 20 ml of 0.9% NaCl solution, carbocaine 2% and adrenaline 1/200,000 **Technique**: 3-mm coleman aspiration cannula **Negative pressure**: manually generated **Volume**: N/A	1. Lipoaspirate centrifuged at 3000 rpm for 3 min.<br>2. Oil removed, and fluid decanted.<br>3. Mechanically emulsified after rinsing, 30 passes through usual connector	N/A	10 ml of nanofat (7 ml) plus PRP (3 ml)	Subcutanoeus under deformities	19G cannulas	18 to 52 yo	N/A	1, 3 and 6 months
Ghareeb et al. 2017 [53]	**Site:** preferably abdomen **Infiltration**:<br>1000 ml of 0.9% saline solution, one ampoule of adrenaline 1 mg/ml, and 15	Lipoaspirate centrifuged at 3000 RPM for 3 min<br>The purified fat underwent mechanical emulsification (30 passes, through usual connector)	N/A	Classic nanofat	Intradermal and subdermal injection of nanofat	27G sharp needles	8 to 48 yo	N/A	5 days, 2 weeks, 1, 3 and 6 months

	ml of lidocaine hydrochloride 2% without adrenaline<br>**Technique**: 3-mm cannula with 50 ml syringe, **Negative pressure**: manual<br>**Volume**: N/A								
Carstens et al. 2017 [54]	**Site**: flanks and abdomen<br>**Infiltration**: N/A<br>**Technique:** N/A<br>**Negative pressure:** N/A<br>**Volume**: 250-350ml,	1.Lipoaspirate washed three times with sterile Lactated Ringer's Solution<br>2.Collagenase added - 200 CDU/ml of total volume, 40 min incubation at 38 °C and 150 rpm.<br>3. Human serum albumin was added (2.5% solution v/v)<br>4. centrifuged, 10 min at 800 g.<br>5. Pellet resuspended in 15 ml Hartmann solution	2,37 - 9,83 x 10^7 viable cells/g of fat	SVF	Subcutaneous injection	19G needle	26 ± 6,22 yo	6,7 ± 4,3 yo	6 months
Bhooshan et al. 2018 [55]	**Site**: N/A<br>**Infiltration**: N/A<br>**Technique**: 3 mm mirrored triport Coleman's cannula<br>**Negative pressure:** manual<br>**Volume:** N/A	Lipoaspirate mechanically emulsified by 30-35 passes through triport connector	N/A	Classic Nanofat	Intralesional	27G needle	32.2 ± 12 yo	3 to 204 months (17 years); 79.4% - scars <5 year 20.6% - scars > 5 years	3 months
Gu et al. 2018 [56]	**Site**: periumbilically<br>**Infiltration**: 20 mL of lidocaine, 0.5%, and 1 mL of 1:1000 epinephrine per 1000 mL of saline<br>**Technique**: 3mm multihole aspiration cannula to a 20-mL syringe, **Negative pressure**: manual by retracted plunger<br>**Volume**: N/A	1. Saline rinsing and filtering,<br>2. Centrifuged at 3000 rpm for 3 min<br>3. The oil layer was decanted, and the aqueous component drained.<br>4. For mechanical emulsification, through connected to the Tulip transfer connector with three 1.4-mm holes 30 passes<br>5. centrifuged again at 3000 rpm for 3 min	N/A	Condensed nanofat	Intradermally, after 18G needle introduced to break underlying adhesions of the scar. Volume restored subdermally with fat graft combined with condensed nanofat through	29G needle/ 1.2 mm blunt cannula	21-62 yo, mean 38.25 yo;	3 to 26 years (mean formati on time, 7.45 years)	6 months

					a blunt 1.2-mm cannula				
Lee et al. 2018 [57]	**Site**: abdomen.<br>**Infiltration:** N/A<br>**Technique**: 3mm blunt-tipped cannula. Liposuction kit used (Lipokit).<br>**Negative pressure**: N/A<br>**Volume**: 50 ml.	1. Centrifugation at 3500 RPM for 4 min.<br>2. Discarding the lower layer (20ml left)<br>3. Adding collagenase type II and incubation for 30 min in 37 °C (MaxSTEM kit)<br>4. Centrifugation at 3500 RPM for 3 min<br>5. Wash in Hartmann solution with 5% dextrose saline and gentamicin<br>6. Step 5 and 6 repeated 3 times	5,9 x 10^7 cells per mL	2 ml of SVF	Subcutaneous and intradermal, no more than 5 ml/case	N/A	Study 1: 14-64 yo (37,47 ± 13,2)<br>Study 2: 19-65 (35,8 ± 14	Study 1: 3-240 mo (22,3 ± 51,8)<br>Study 2: 0-18 mo (6,53 ± 4,47)	6 months
Uyulmaz et al. 2018 [58]	**Site**: chosen individually.<br>**Infiltration:** 900 mL NaCl 0.9%, 0.25 mL adrenaline (1 mg/ mL), 20 mL of lidocaine (20 mg/mL).<br>**Technique**: Tonnard 2.4 mm × 20 cm cannula with sharp side 1 mm holes<br>**Negative pressure**: N/A<br>**Volume**: 10-800 ml, mean 165	1. Wash with isotonic saline solution.<br>2. Mechanically emulsification 30 passes (2.4 mm Tulip connector)<br>3. Filtration through a nylon cloth with 0.5 mm pore size	N/A	Classic nanofat 1 to 25 mL (mean, 4.6 mL)	Injected intralesionally or intradermally	24, 25 or 27G sharp needles	15 to 64 years (mean, 42 years)	15 to 40 years (mean, 5.8 years	155 ± 49 days (range, 87-312 days)
Abou Eitta et al. 2019 [62]	**Site**: abdomen, thighs, buttocks<br>**Infiltration**: modified Klein's solution<br>**Technique**: blunt-tipped cannula, 60 ml syringe<br>**Negative pressure**: probably manually<br>**Volume**: ~ 50 ml	1. Lipoaspirate washed with PBS and antibiotics/ antimycotic<br>2. Gravitational decanting and discarding of infranatant<br>3. Steps 2 and 3 repeated 6 times<br>4. Collagenase type IA and incubation for 37 °C for 1 hour<br>5. Infranatant with SVF aspirated and DMEM with 10% FBS added to inactivate the collagenase<br>6. Centrifugation at 300g for 10 min<br>7. SVF pellets collected with PBS, filtration with 100um cell strainer	Average: 6x10^6 cells	SVF suspended in 1 ml PBS	Reported as injected intradermally (but „underneath atrophic scars")	N/A	20 to 45 yo (mean 33.20 ± 6.51)	N/A	1, 2 and 3 months

		8. Centrifugation at 300g for 5 min<br>9. Optional RBC lysis buffer at room temperature for 5 min; then centrifuged again for 5 min.<br>10. Pellet washed twice with PBS<br>11. Resuspended in 1mL PBS, ready for injection							
Malik et al. 2019 [67]	**Site**: abdomen, thighs<br>**Infiltration**: Ringer lactate with epinephrine 1:400,000<br>**Technique**: liposuction cannula (no details) connected with a 10 ml syringe<br>**Negative pressure:** manually, plunger pulled back only a few ml (?)<br>**Volume**: 25-65 ml, mean 34 ml	1. Gravitational decanting, oil and aqueous phase discarded<br>2. 0.075% collagenase added, incubated for 30 min at 37 °C with agitation<br>3. Centrifugation at 1200 RPM for 5 min.<br>4. Collection of SVF from the pellet	N/A	SVF, 10 ml?	Under scar – subcutaneous	Blunt infiltration cannula	22-45 yo, mean 32.1	N/A	1 and 6 months
Jan et. Al 2019 [59]	**Site**: abdomen, lateral thigh, and/or the gluteal region.<br>**Infiltration**: 0.9% saline 1000 mL, lidocaine 30 mL, and 1 mL of 1:1000 epinephrine<br>**Technique**: 3-mm cannula with multiple sharp side holes of 1 mm attached to a 20-mL syringe. **Negative pressure:** plunger back by 2 mL<br>**Volume**: N/A	1. Lipoaspirate rinsed with 0.9% saline<br>2. Emulsification by 30 syringe-syringe passes (unknown connector)	N/A	Classic nanofat	Selective intradermal or subdermal pre-tunneling. Fanwise pattern with a 1-mL syringe until the skin blanched or yellowish	18G	22.25 ± 5.79 y	>1yo	6 months
Shalaby et al. 2020 [63]	**Site**: preferably abdomen or flanks/inner knees<br>**Infiltration**: 500 ml of 0.9% saline solution, adrenaline 0.5 mg/ml, and 20 ml of lidocaine hydrochloride 2% without adrenaline<br>**Technique**: Unknown cannula, 20ml syringe	1) Lipoaspirate washed with Lactated Ringer, incubated 3 min<br>2) centrifuged at 3000 RPM 3 min<br>3) Middle layer preserved<br>4) Mechanically emulsified by shifting s by 30 passes through 2.4 mm tulip connector<br>5) Another 30 passes with using (1.4 mm tulip connector	N/A	Nanofat or nanofat + PRP	Superficial intradermal nanofat and additional subdermal injection	28G needle and 22-23G cannula	32.8 ± 11.2 yo (nanofat + PRP); 26.5 ± 9.1 yo (nanofat)	6.8 ± 7.6 yo (nanofat + PRP); 3.3 ± 2.7 (nanofat)	3 months

	**Negative pressure**: retracted plunger<br>**Volume**: N/A	6) 600 um nanofat filtration							
Pallua et al. 2020 [52]	**Site**: preferably abdomen and/or other<br>**Infiltration**: adrenaline suspended in saline solution in a ratio of 1:200,000<br>**Technique**: blunt-tipped thin cannula, with a diameter of 2 mm, and 4 orifices, each gauge measuring 600um<br>**Negative pressure:** N/A<br>**Volume**: N/A	1. lipoaspirate is centrifuged at 1200 g for 3 min<br>2. The oily and watery layers removed<br>3. Manual emulsification by 30 passes, usual connector<br>4. Centrifugation at 1200 g for 3 min<br>5. Middle layer preserved	N/A	Condensed nanofat, in 1 case supplemented with PRP	Various author's preparation of recipient site before injections. Subcutaneous and/or intradermal	27G cannula.	41,33 ± 10,53	N/A	6 - 12 months

AFT – autologous fat transfer; N/A – not available; PRP – platelet-rich plasma; RCF – relative centrifugal force; RPM – revolutions per minute; SVF – stromal vascular fractions; yo – years old; mo – months old
